# Prognostic impact of changes in left ventricular ejection fraction and wall motion score index in patients with myocardial infarction

**DOI:** 10.3389/fcvm.2025.1530006

**Published:** 2025-05-14

**Authors:** Min-Wook Bae, Seong-guen Moon, Kyung-Tae Jung, Won-Ho Kim, Sang-Hyun Park, Jihun Ahn, Jin-Yong Hwang, Seok Kyu Oh, Seung Ho Hur, Myung Ho Jung, Kyu-Sun Lee

**Affiliations:** ^1^ Department of Internal Medicine and Cardiovascular Center, Daejeon Eulji University Hospital, Daejeon, Republic of Korea; ^2^ Department of Internal Medicine, Gyeongsang National University School of Medicine, Gyeongsang National University Hospital, Jinju, Republic of Korea; ^3^ Department of Cardiology, Wonkwang University Hospital, Iksan, Republic of Korea; ^4^ Department of Cardigology, Keimyung University Dongsan Medical Center, Daegu, Republic of Korea; ^5^ Department of Cardiology, Chonnam National University Hospital, Chonnam National University Medical School, Gwangju, Republic of Korea

**Keywords:** acute myocardial infarction, left ventricular ejection fraction, wall motion abnormality, LV reverse and adverse remodeling, Korean acute myocardial infarction registry acute myocardial infarction, intravascular imaging-guided PCI, beta-blocker therapy, LV remodeling

## Abstract

**Background:**

The prognostic significance of changes in left ventricular ejection fraction (LVEF) and wall motion score index (WMSI) in patients with myocardial infarction remains unclear.

**Methods:**

This study evaluated whether changes in LVEF and WMSI can predict clinical outcomes and LV remodeling in post-AMI patients. Using data from the Korea Acute Myocardial Infarction Registry-National Institutes of Health (KAMIR-NIH), 3,510 AMI patients who underwent percutaneous coronary intervention (PCI) were retrospectively analyzed. LVEF and WMSI were assessed via echocardiography at baseline and one-year post-PCI. The primary outcome was major cardiovascular adverse events (MACE), a composite of all-cause death, recurrent myocardial infarction (MI), and rehospitalization for heart failure at three years.

**Results:**

Among 3,510 AMI patients, 1,561 (44.5%) showed improvement in both LVEF and WMSI at one year after PCI, 1,150 (32.8%) experienced improvement in either LVEF or WMSI, while 799 (22.8%) had deterioration in both. The incidence of MACE was significantly lower in patients with improvement in both LVEF and WMSI (7.8% vs. 12.5% vs. 17.1%, *P* < 0.001). These patients also exhibited the highest rate of LV reverse remodeling and the lowest rate of adverse remodeling. Both the random forest and logistic regression models identified changes in LVEF and WMSI as significant predictors of MACE and LV remodeling.

**Conclusion:**

In AMI patients, improvement in both LVEF and WMSI post-PCI was associated with a lower risk of MACE and a higher likelihood of LV reverse remodeling. These findings highlight the prognostic value of LVEF and WMSI changes in guiding long-term management strategies.

## 1 Introduction

Acute myocardial infarction (AMI) remains a leading cause of morbidity and mortality worldwide. Timely reperfusion through primary percutaneous coronary intervention (PCI) is a crucial step in salvaging the myocardium and improving survival (1). However, despite advancements in PCI technology and adjunctive medical therapies, a significant proportion of AMI patients experience left ventricular (LV) remodeling (2–5). A previous study reported that nearly 48% of patients with ST-elevation myocardial infarction (STEMI) experienced post-infarct LV remodeling within the first 12 months of follow-up (6). LV adverse remodeling refers to the maladaptive structural and functional changes in the left ventricle following AMI (7, 8). It is characterized by progressive LV dilation, increased LV end-diastolic and end-systolic volumes, wall thinning, and impaired contractility. These changes contribute to worsening heart failure symptoms, increased arrhythmic risk, and higher mortality (9, 10).

Echocardiography parameters, such as LV ejection fraction (LVEF), wall motion score index (WMSI), and LV end-systolic and end-diastolic volume, have been widely used to assess LV remodeling and predict long-term prognosis in AMI patients. Among various echocardiographic parameters, we focus on LVEF and WMSI because these are the parameters most commonly used by clinicians to evaluate the effectiveness of PCI and pharmacological therapy after AMI. These parameters can be recovered through revascularization via PCI and guideline-directed medical therapy. Given the dynamic nature of LVEF and WMSI, assessing these parameters at follow-up may provide better prognostic insights than a single assessment at baseline. This study aimed to investigate whether serial changes in LVEF and WMSI can predict clinical outcomes and LV remodeling in AMI patients who underwent PCI.

## 2 Methods

### 2.1 Study population and data collection

The Korea Acute Myocardial Infarction Registry-National Institutes of Health (KAMIR-NIH) is the first nationwide, prospective, multicenter registry of Korean patients with AMI, launched in November 2005**.** This study included patients with AMI who underwent PCI at 15 medical institutions in Korea between November 2011 and December 2015. All participants provided written informed consent before enrollment in KAMIR-NIH, and their clinical data were prospectively recorded. Patients were followed up at 6, 12, 24, and 36 months to monitor adverse events after PCI, following the KAMIR cohort protocol (11). The diagnosis of AMI was based on clinical manifestations, electrocardiography, and serum levels of cardiac biomarkers [creatinine kinase (CK)-MB and troponin I] and was classified as either STEMI or non-ST-segment elevation myocardial infarction (NSTEMI) (12–14). Transthoracic echocardiography was performed by a clinical echocardiography fellow or a well-trained sonographer. LV volume and LV ejection fraction were calculated using the apical orthogonal (2ch & 4ch) via the Biplane Simpson method. Segment contractility scoring based on segment excursion and wall thickening was defined as normal, hypokinetic, akinetic, dyskinetic, or aneurysm. The wall motion score index (WMSI), a surrogate marker of regional wall motion abnormalities, was calculated by dividing the sum of the wall motion score by the number of visualized segments according to the American Society of Echocardiography guidelines (15–17). A higher WMSI indicated more severe abnormal wall motion. Finally, 3,510 patients with AMI who underwent serial transthoracic echocardiography were retrospectively analyzed. The study protocol was approved by the Institutional Review Board (IRB) of Eulji University Hospital (IRB No. 2024-04-013) and adhered to the ethical guidelines of the 2013 Declaration of Helsinki.

### 2.2 Study definition and outcome

The primary outcome was major adverse cardiovascular events (MACE), defined as a 3-year composite of all-cause mortality, recurrent MI, or rehospitalization for HF. Secondary outcomes included major adverse cardiovascular or cerebrovascular events (MACCE), a 3-year composite of all-cause mortality, recurrent MI, any repeat revascularization, ischemic stroke or stent thrombosis (ST), as well as LV remodeling. Changes in LV systolic function and WMAs were assessed by calculating differences in LVEF and WMSI between baseline and one year after the index PCI. LVEF and WMAs improvement is defined as an increase in LVEF and a decrease in WMSI at 12 months compared to baseline values. Post-infarct LV adverse remodeling was defined as LV dilatation with an LV-end diastolic volume (LVEDV) increase of ≥20% compared to baseline value (18–20). LV reverse remodeling was defined as a ≥15% reduction in LV-end systolic volume (LVESV) and an improvement of LVEF ≥ 10% compared to baseline (21). Both LV reverse and adverse remodeling were assessed at the one-year follow-up after PCI.

### 2.3 Statistical analysis

Categorical data were compared using the chi-squared or Fisher's exact test, as required. Continuous data are presented as mean ± standard deviation or medians (25th‒75th percentiles), and group differences were compared using Student's t-test or the Mann–Whitney test. Analysis of variance (ANOVA) or the Kruskal‒Wallis test was used to compare three or more independent groups. To identify factors associated with clinical outcomes and LV remodeling, we applied both a random forest model and a logistic regression model, which are commonly used for predicting outcomes and assessing variable importance. The random forest model, an ensemble learning method, was used to capture complex, non-linear relationships between predictors and outcomes. This model allowed for the assessment of variable importance in predicting MACE and LV remodeling while minimizing overfitting. The model was built using bootstrap aggregation (bagging) with 500 decision trees, and variable importance was ranked based on the mean decrease in Gini impurity. To complement this analysis, a multivariable logistic regression model was used to evaluate the independent associations between changes in LVEF and WMSI and clinical outcomes or LV remodeling. Covariates were selected based on clinical relevance and statistical significance in univariable analysis (*P* < 0.05) and subsequently incorporated into the multivariable model. The Hosmer-Lemeshow goodness-of-fit test and the area under the receiver operating characteristic (ROC) curve (AUC) were used to assess model performance. The cumulative incidence of primary outcomes was calculated using the Kaplan–Meier estimates at 3 years, and the log-rank test was used to estimate group differences. Cox proportional hazard regression assessed the hazard ratio (HR) and 95% confidence interval (CI) of 3-year primary outcomes. The following variables were included for adjustment: age, sex, body mass index, diabetes mellitus, hypertension, chronic kidney disease, current smoking, prior revascularization, baseline blood pressure, heart rate, ejection fraction, clinical diagnosis, coronary artery disease status, coronary artery lesion type, revascularization status, and pharmacotherapy including P2Y12 inhibitors, beta-blockers, and ACE inhibitors therapy. Inter-operator variability was assessed using hospital-based differences as a proxy. We estimated hospital-related variability as an indicator of inter-operator variability by applying linear regression and calculating R² values. All tests were two-tailed, and a *P* value < 0.05 was considered statistically significant. All statistical analyses were performed using SPSS version 26.0 (IBM SPSS Statistics, Chicago, Illinois, USA) and Python (version 3.8, Python Software Foundation).

## 3 Results

### 3.1 Patient classification according to the changes in LVEF and WMSI and baseline characteristics

**Figure 1:**
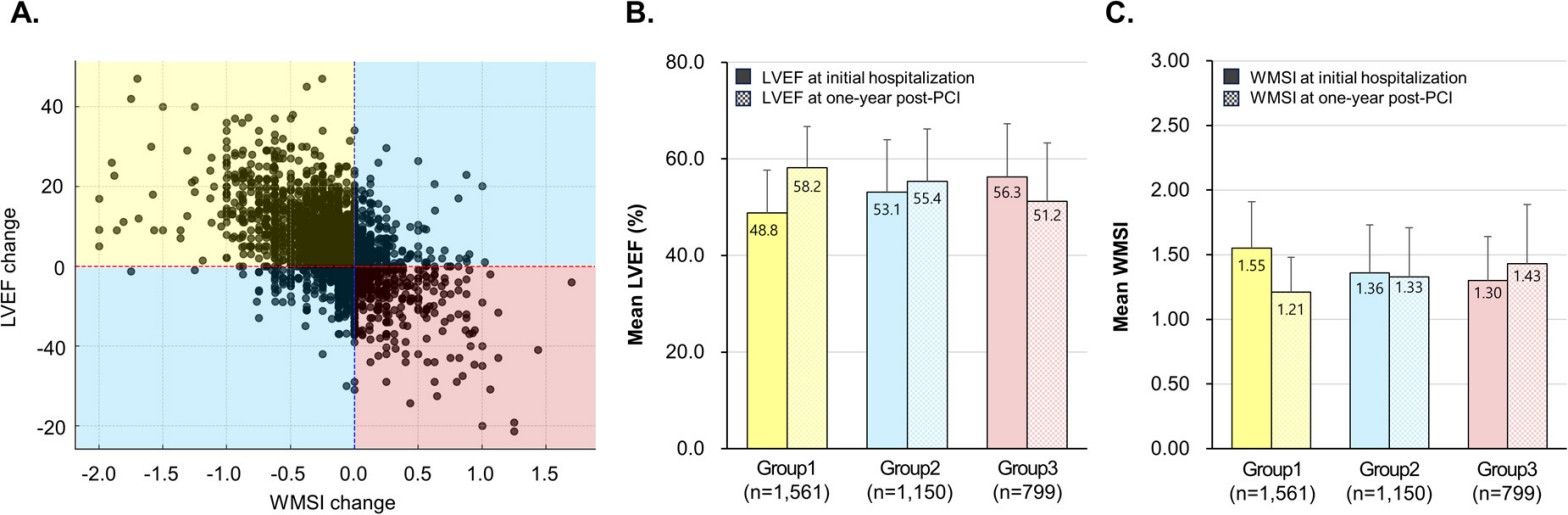
Patient classification based on LVEF and WMSI changes. The scatter plot showed the relationship between changes in LVEF and WMSI, and patients were classified into three groups based on LVEF and WMSI changes **(A)**, mean LVEF **(B)**, and mean WMSI **(C)** from initial hospitalization to one year post-PCI in AMI patients. LVEF, left ventricular ejection fraction; WMSI, wall motion score index; PCI, percutaneous coronary intervention.

LVEF has a negative linear correlation with WMSI (Pearson's correlation coefficient = −0.73; *P* < 0.001) (Supplementary Figure S1). Furthermore, changes in LVEF show a negative correlation with changes in WMSI (Pearson's correlation coefficient = −0.54; *P* < 0.001) while also demonstrating that patients can be classified into three distinct types based on these changes (Figure 1). Among the 3,510 patients with AMI, 1,561 (44.5%) showed both an increase in LVEF(from 48.8% to 58.2%) and a decrease in WMSI (from 1.55 to1.21) at the 1-year follow-up compared to baseline. Another 1,150 patients (32.8%) exhibited either an increase in LVEF (from 53.1% to 55.4%) or an alleviated WMSI (from 1.36 to 1.33). In contrast, 799 patients (22.8%) experienced a decline in LV systolic function (from 56.3% to 51.2%) along with a worsening of WMSI (from 1.30 to 1.43). To quantify the extent of variation in the measurement of LVEF and WMSI, we used hospital-related variability in LVEF and WMSI as a proxy for inter-operator variability by applying linear regression and computing R² values There is no statistically significant difference between the baseline and 12-month inter-operator variability for LVEF and WMSI (t-statistic = 0.857; *P*-value = 0.549). LVEF and WMSI measurements remain consistent across hospitals, indicating operator consistency (Supplementary Figure S2).

Table 1 presents the baseline characteristics of patients who showed improvement in both LVEF and WMSI compared to those who had improvement in either LVEF or WMSI alone or no improvement in both. Compared to patients with either LVEF or WMSI improvement alone or no improvement in both, those with improvement in both LVEF and WMSI had a lower proportion of males and significantly lower prevalence of hypertension, prior myocardial infarction, and previous revascularization. Patients who experienced improvement in both LVEF and WMSI had a higher proportion of STEMI, lower baseline LVEF, and higher baseline WMSI at the time of admission, compared to those with either LVEF or WMSI improvement alone or no improvement in both. Additionally, the symptom-to-balloon time was significantly shorter in patients with both LVEF and WMSI improvement than in those with only one or neither improvement. These patients also had a lower rate of IVUS-guided PCI, higher NT-proBNP levels, and were significantly more likely to receive beta-blockers and ACE inhibitors.

**Table 1:** Baseline patient characteristics in patient groups according to changes in LV ejection fraction and wall motion score index.

Characteristics	Total population (*N* = 3,510)	LVEF- WMSI improvement (*N* = 1,561)	LVEF-WMSI Non-improvement (*N* = 1,949)	*P*-value
Age, years	61.7 ± 11.9	61.6 ± 11.7	62.0 ± 11.9	0.337
Male	2,720 (77.5)	1,182 (75.7)	1,538 (78.9)	0.025
Body mass index, kg/m^2^	24.2 ± 3.3	24.0 ± 3.2	24.4 ± 3.3	0.260
Hypertension	1,621 (46.2)	674 (43.2)	947 (48.6)	0.002
Diabetes mellitus	898 (25.6)	394 (25.2)	504 (25.9)	0.697
Dyslipidemia	399 (11.4)	181 (11.6)	218 (11.2)	0.708
Prior myocardial infarction	170 (4.8)	52 (3.3)	118 (6.1)	<0.001
Prior revascularization		87 (5.6)	176 (9.0)	<0.001
Prior heart failure	31 (0.9)	10 (0.6)	21 (1.1)	0.205
Prior stroke	151 (4.7)	71 (4.5)	98 (5.0)	0.527
Smoking status				0.827
Never smoker	1,254 (35.7)	563 (36.1)	691 (35.5)	
Ex-smoker	669 (19.1)	297 (19.0)	372 (19.1)	
Current smoker	1,505 (42.9)	661 (42.3)	884 (43.3)	
Killip classification				0.352
Class I	2,800 (79.8)	1,234 (79.1)	1,566 (80.3)	
Class II–III	710 (20.2)	327 (20.9)	383 (19.7)	
SBP at admission		130.3 ± 28.4	129.2 ± 28.8	0.242
DBP at admission		79.5 ± 17.8	78.8 ± 18.5	0.108
Heart rate at admission		78.8 ± 18.5	76.5 ± 18.2	<0.001
Presentations				0.002
STEMI	2,023 (57.6)	947 (60.7)	1,080 (55.4)	
NSTEMI	1,487 (42.4)	614 (39.3)	869 (44.6)	
LVEF at admission, %	51.9 ± 10.5	48.8 ± 8.9	54.4 ± 11.0	<0.001
WMSI at admisison	1.43 ± 0.38	1.55 ± 0.36	1.33 ± 0.36	<0.001
Initial CAD status				0.185
LM disease	114 (3.2)	47 (3.0)	77 (4.0)	
One vessel	1,716 (48.9)	783 (50.2)	933 (47.9)	
Two vessel	1,051 (29.9)	477 (30.6)	574 (29.5)	
Three vessel	619 (17.6)	254 (16.3)	365 (18.7)	
Imaging-guided PCI				
IVUS use	949 (27.0)	396 (25.4)	553 (28.4)	0.047
OCT use	105 (3.0)	48 (3.1)	57 (2.9)	0.842
FFR-guided PCI	56 (1.6)	28 (1.8)	28 (1.4)	0.419
ACC/AHA lesion classification				0.086
Type A	28 (0.8)	17 (0.7)	11 (1.2)	
Type B1	443 (12.6)	338 (13.1)	105 (11.3)	
Type B2	1,133 (32.3)	847 (32.8)	286 (30.7)	
Type C	1,906 (54.3)	1,377 (53.4)	529 (56.8)	
Revascularization strategy				0.208
Culprit-lesion-only PCI	2,850 (81.2)	1,282 (82.1)	1,568 (80.5)	
Multivessel PCI	660 (18.8)	279 (17.9)	381 (19.5)	
PCI procedures				0.583
Index procedure PCI	3,135 (89.3)	1,389 (89.0)	1,746 (89.6)	
Staged PCI	375 (10.7)	172 (11.0)	203 (10.4)	
Symptom-to-balloon time (hr)	7.7 (2.9–27.2)	6.5 (2.8–25.2)	8.5 (2.9–30.2)	0.025
Laboratory findings				
Hemoglobin (mg/dl)	14.2 ± 1.9	14.2 ± 1.9	14.1 ± 1.9	0.850
Platelet (×10^3^)	234.9 ± 66.6	238.9 ± 69.4	231.8 ± 64.0	0.256
Creatinine	1.0 ± 0.8	1.0 ± 0.9	1.0 ± 0.7	0.941
CK-MB	125.4 ± 151.1	114.6 ± 133.9	134.1 ± 163.1	<0.001
TnI	53.8 ± 112.3	51.3 ± 133.7	55.7 ± 91.7	0.286
TG	141.6 ± 120.8	138.0 ± 119.5	144.4 ± 121.9	0.126
HDL-C	43.2 ± 11.8	43.4 ± 11.6	42.6 ± 12.4	0.033
LDL-C	116.8 ± 39.0	117.9 ± 39.2	115.8 ± 38.8	0.140
NT-proBNP	1,396.0 ± 3,878.0	15,518. ± 4,191.9	1,271.25 ± 3,604.2	0.076
HbA1C	6.4 ± 1.4	6.4 ± 1.4	6.4 ± 1.4	0.726
Medication				
Clopidogrel	2,585 (73.6)	1,140 (73.0)	1,445 (74.1)	0.464
Ticagrelor or prasugrel	1,378 (39.3)	618 (39.6)	760 (39.0)	0.728
Calcium-channel blocker	148 (4.2)	64 (4.1)	84 (4.3)	0.800
Beta-blocker	3,049 (86.9)	1,393 (89.2)	1,656 (85.0)	<0.001
ACE inhibitors	1,825 (52.0)	875 (56.1)	950 (48.7)	<0.001
Angiotensin receptor blocker	1,098 (31.3)	813 (31.5)	285 (30.6)	0.621
Statin	3,356 (95.6)	1,495 (95.8)	1,861 (95.5)	0.740

LV, left ventricular; WMA, wall motion abnormality; AMI, acute myocardial infarction; STEMI, ST-elevation myocardial infarction; NSTEMI, non-ST elevation myocardial infarction; LVEF, left ventricular ejection fraction; WMSI, wall motion score index; CAD, coronary artery disease; LM, left main; PCI, percutaneous coronary intervention; IVUS, intravascular ultrasound; OCT, optical coherence tomography; FFR, fractional flow reserve; LAD, left anterior descending artery, LCX, left circumflex artery; RCA, right coronary artery; ACC/AHA, American college of cardiology/American heart association; CK-MB, creatinine kinase MB, TnI, troponin I; TG, triglyceride; HDL-C, high-density lipoprotein cholesterol; LDL-C, low-density lipoprotein cholesterol; NT-proBNP, n-terminal pro-B type natriuretic peptide; HbA1C, hemoglobin A1C; ACE, angiotensin-converting enzyme.

### 3.2 Clinical outcomes based on changes in LVEF and WMSI

**Figure 2:**
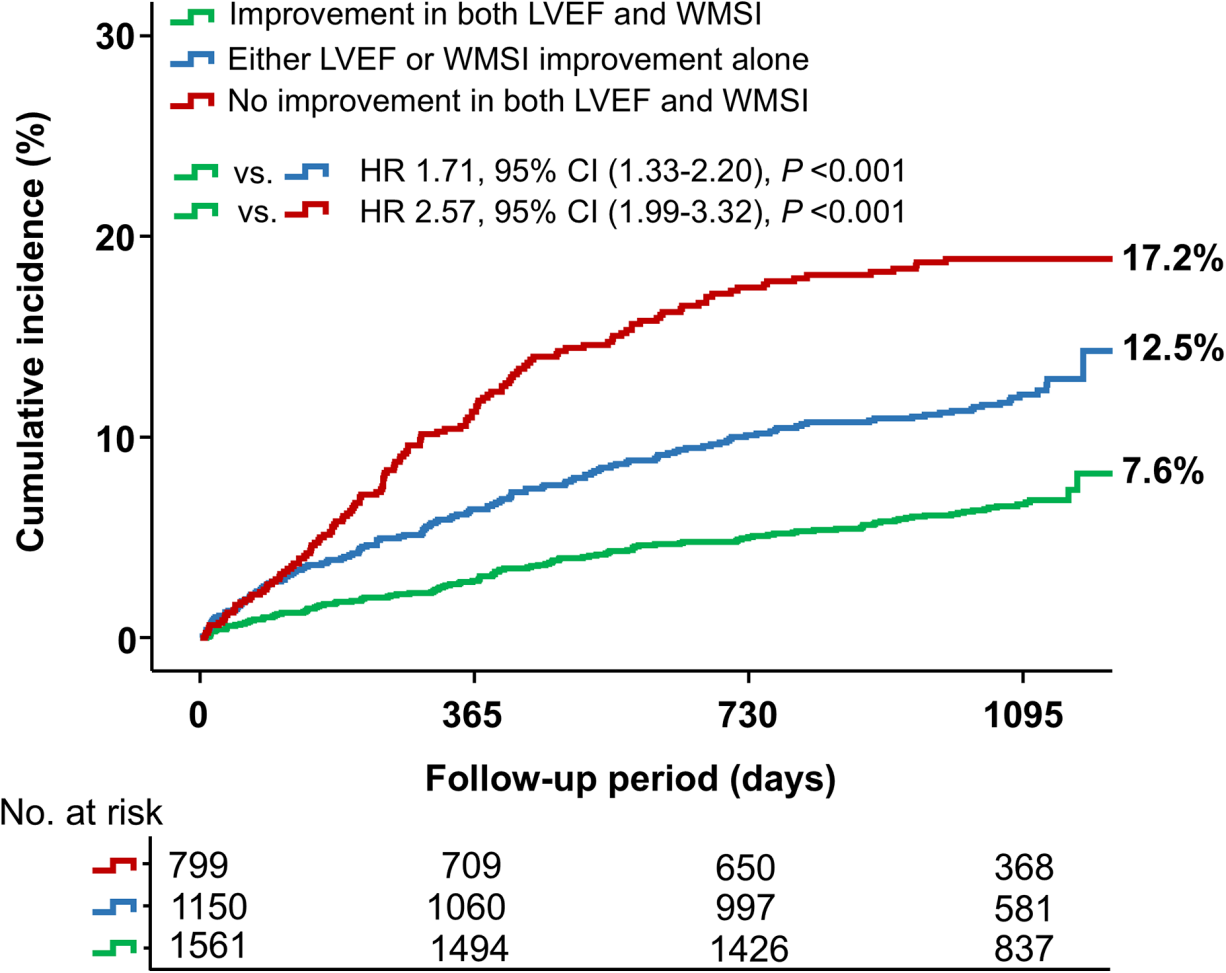
Major adverse cardiovascular events (MACE) based on changes in LVEF and WMSI. The Kaplan–Meier curve illustrates the cumulative incidence of MACE based on changes in LVEF and WMSI among patients. LVEF, left ventricular ejection fraction; WMSI, wall motion score index; HR, hazard ratio.

Figure 2 and Table 2 present the clinical outcomes according to changes in LVEF and WMSI. Patients who experienced improvement in both LVEF and WMSI had a significantly lower risk of MACE (a composite of all-cause mortality, recurrent MI, and HF-related rehospitalization) compared to those with improvement in either LVEF or WMSI alone or no improvement in both. Specifically, the hazard ratio (HR) for MACE was 1.71 (95% CI: 1.33–2.20, *P* < 0.001) when comparing patients with improvement in both LVEF and WMSI to those with improvement in either LVEF or WMSI alone. Similarly, the HR was 2.57 (95% CI: 1.99–3.32, *P* < 0.001) when comparing patients with improvement in both LVEF and WMSI to those with no improvement in either parameter. Additionally, the incidence of MACCE, a composite of all-cause mortality, recurrent MI, any repeat revascularization, ischemic stroke, or stent thrombosis, was also significantly lower in this group (15.0% vs. 20.2% vs. 21.7%, *P* < 0.001). These results were driven by a lower incidence of all-cause death (1.6% vs. 2.9% vs. 4.0%, *P* = 0.002), recurrent MI (3.7% vs. 5.2% vs. 5.6%, *P* = 0.047), HF-related rehospitalization (3.3% vs. 5.3% vs. 9.8%, *P* < 0.001), any repeat revascularization (10.7% vs. 13.7% vs. 15.4%, *P* = 0.003), and stent thrombosis (0.4% vs. 1.2% vs. 1.1%, *P* = 0.063) in patients who showed improvement in both LVEF and WMSI.

**Table 2:** Clinical outcomes in patients based on LVEF and WMSI changes.

Outcomes-no.(%)	Both improved (*N* = 1,561)	Either improved (*N* = 1,150)	Neither improved (*N* = 799)	*P*-value
MACE^a^	121 (7.6)	144 (12.5)	137 (17.2)	<0.001
MACCE^b^	234 (15.0)	232 (20.2)	173 (21.7)	<0.001
LV adverse remodeling^c^	191 (12.2)	190 (16.5)	173 (21.7)	<0.001
LV reverse remodeling^d^	252 (16.1)	31 (2.7)	0 (0.0)	<0.001
All-causes of death	25 (1.6)	33 (2.9)	32 (4.0)	0.002
Recurrent MI	57 (3.7)	60 (5.2)	45 (5.6)	0.047
Rehospitalization for HF	51 (3.3)	61 (5.3)	78 (9.8)	<0.001
Repeat revascularization	167 (10.7)	157 (13.7)	123 (15.4)	0.003
Ischemic stroke	34 (2.2)	26 (2.3)	12 (1.5)	0.455
Stent thrombosis	7 (0.4)	14 (1.2)	9 (1.1)	0.063

LVEF, left ventricular ejection fraction; WMSI, wall motion score index; MACE, major adverse cardiovascular events; MACCE, major adverse cardiovascular and cerebrovascular events; MI, myocardial infarction; HF, heart failure.

^a^ composite of all-cause mortality, recurrent MI, and rehospitalization due to HF.

^b^ composite of all-cause mortality, recurrent MI, repeat revascularization, ischemic stroke, or stent thrombosis.

^c^ defined as LV dilatation with an LV-end diastolic volume (LVEDV) increase of ≥20% compared to baseline value.

^d^ defined as a ≥ 15% reduction in LV-end systolic volume (LVESV) and improvement of LVEF ≥ 10% from baseline to one-year follow-up after PCI.

**Figure 3:**
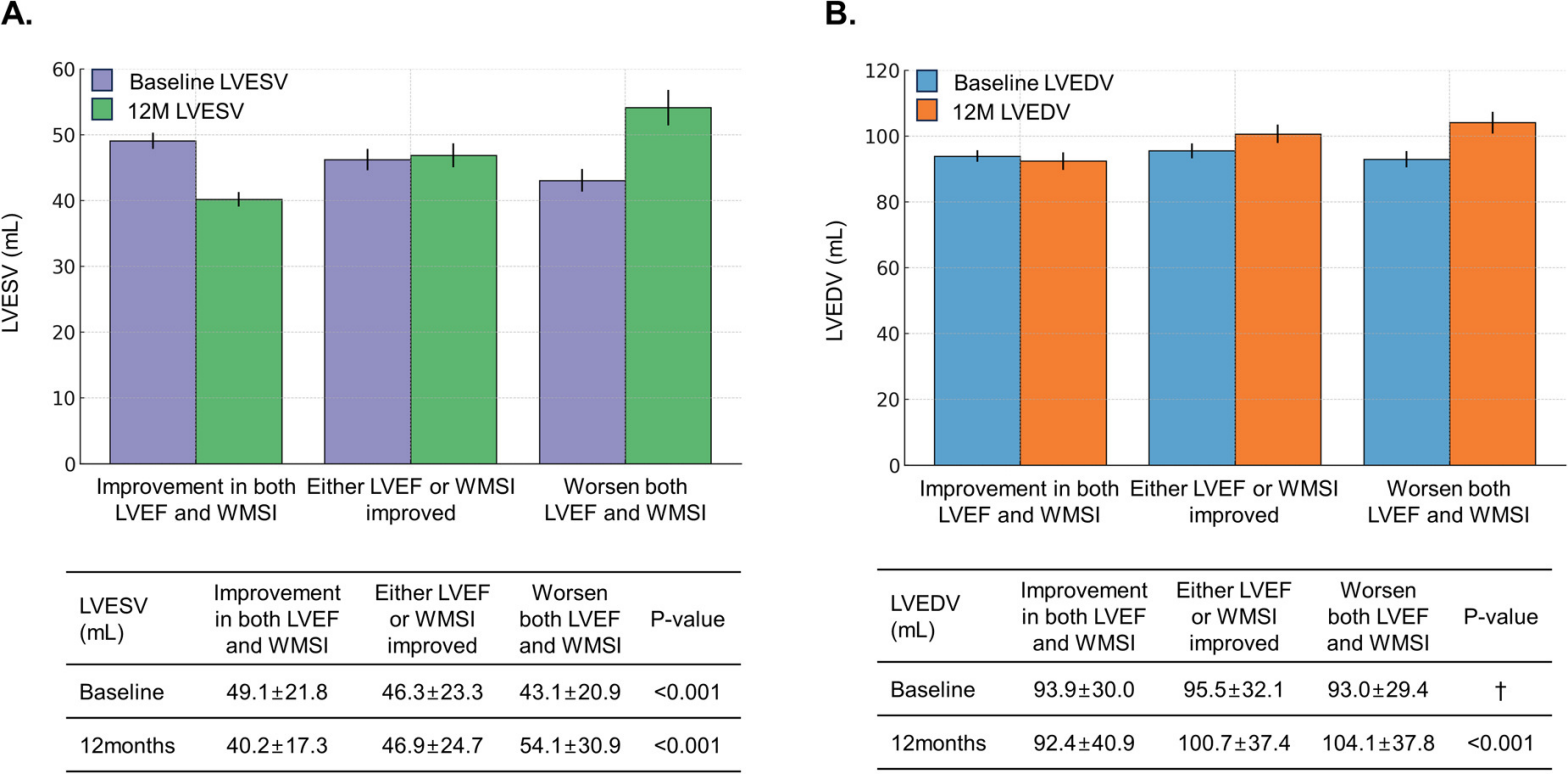
Changes in LV end-systolic volume (LVESV) and LV end-diastolic volume (LVEDV) according to LVEF and WMSI changes. The bar graph depicts changes in LV end-systolic volume (LVESV) **(A)** and LV end-diastolic volume (LVEDV) **(B)** at initial hospitalization and one year post-PCI, based on LVEF and WMSI changes among patients. †means statistically insignificant. LVEF, left ventricular ejection fraction; WMSI, wall motion score index; PCI, percutaneous coronary intervention.

Furthermore, these patients had higher rates of LV reverse remodeling (16.1% vs. 2.7% vs. 0.0%, *P* < 0.001) and lower rates of LV adverse remodeling (12.2% vs. 16.5% vs. 21.7%, *P* < 0.001) than those with either LVEF or WMSI improvement alone or no improvement in both. Examining changes in LV end-systolic volume (LVESV) and LV end-diastolic volume (LVEDV)−key indicators of LV remodeling−in relation to changes in LVEF and WMSI, we observed that worsening LVEF, WMSI, or both was associated with a significant increase in LVESV and LVEDV at 12 months post-AMI (Figure 3). Pairwise comparison of ROC curves revealed that changes in LVEF and WMSI more precisely predicted LV reverse remodeling more precisely than baseline LVEF or WMSI, regardless of baseline LVEF (Supplementary Figure S3). Furthermore, changes in LVEF and WMSI predicted LV adverse remodeling more precisely than baseline LVEF or WMSI in patients with baseline LVEF ≤40% (Supplementary Figure S4).

### 3.3 Changes in LVEF and WMSI are predictors of future clinical outcomes

**Figure 4:**
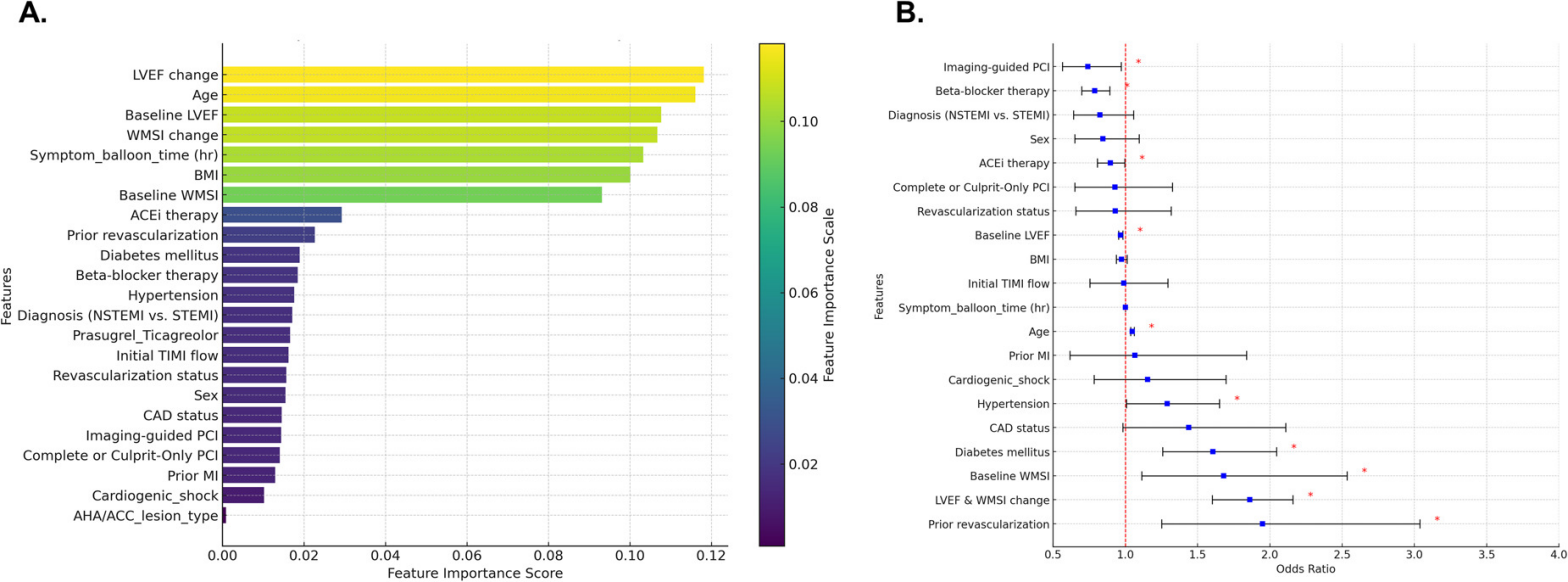
Predicting factors for major adverse cardiovascular events (MACE). Random forest model **(A)** and logistic regression model **(B)** were utilized to identify predictors of MACE, and the results are presented. LVEF, left ventricular ejection fraction; WMSI, wall motion score index; BMI, body mass index; ACEi, Angiotensin-converting enzyme inhibitors; STEMI, ST-elevation myocardial infarction; NSTEMI, non-ST-elevation myocardial infarction; TIMI, thrombolysis in myocardial infarction; CAD, coronary artery disease; PCI, percutaneous coronary intervention; AHA/ACC, American Heart Association and American College of Cardiology.

To identify predictors of MACE, a random forest model was utilized, incorporating clinical factors, echocardiographic parameters, and treatment-related factors. As a powerful ensemble learning method, the random forest model enhances predictive accuracy by combining multiple decision trees. In this model, changes in LVEF and WMSI emerged as the most relevant factors for MACE prediction, along with symptom-to-balloon time, body mass index, diabetes mellitus, hypertension, prior revascularization, beta-blocker therapy, and ACE inhibitor therapy (Figure 4A). Similarly, in the logistic regression model, changes in LVEF and WMSI were also identified as significant predictors of MACE (Figure 4B).

**Figure 5:**
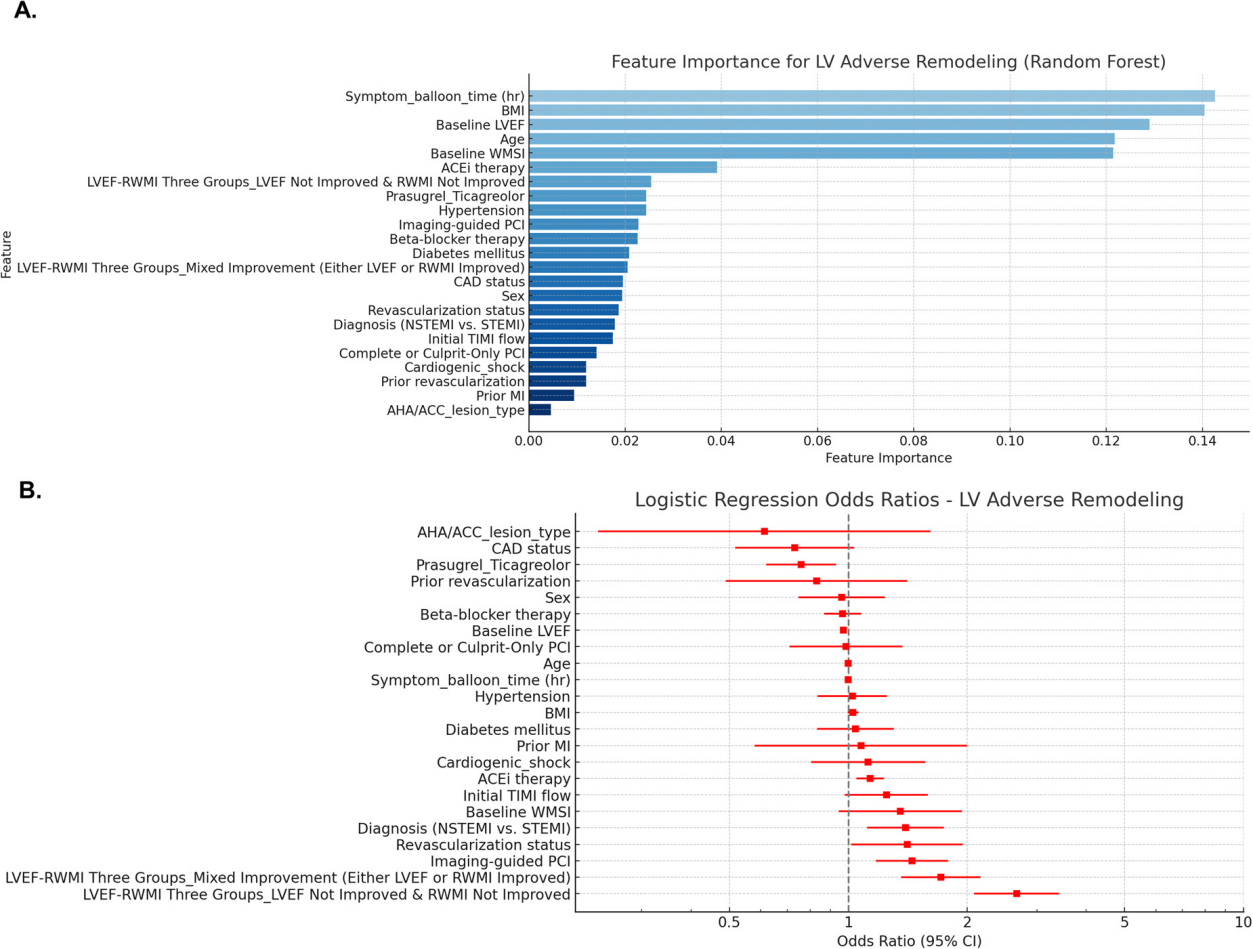
Predicting factors for LV adverse remodeling. Random forest model **(A)** and logistic regression model **(B)** were utilized to identify predictors of LV adverse remodeling, and the results are presented. LVEF, left ventricular ejection fraction; WMSI, wall motion score index; BMI, body mass index; ACEi, Angiotensin-converting enzyme inhibitors; STEMI, ST-elevation myocardial infarction; NSTEMI, non-ST-elevation myocardial infarction; TIMI, thrombolysis in myocardial infarction; CAD, coronary artery disease; PCI, percutaneous coronary intervention; AHA/ACC, American Heart Association and American College of Cardiology.

Changes in LVEF and WMSI are also among the predictors of post-infarct LV remodeling. In the random forest model for predicting LV adverse or reverse remodeling, changes in LVEF and WMSI were among the top-ranked factors, along with symptom-to-balloon time, age, hypertension, diabetes mellitus, pharmacological treatments (beta-blockers and ACE inhibitors), and imaging-guided PCI. Compared to patients with improvement in both LVEF and WMSI, those with either LVEF or WMSI improvement alone or no improvement in both had a higher risk of LV adverse remodeling and a lower likelihood of reverse remodeling (Figure 5 and Supplementary Figure S5).

## 4 Discussion

Using data from a nationwide multicenter registry of Korean patients with AMI, this study found that changes in LVEF and WMSI varied among patients who underwent PCI for AMI. Furthermore, the study demonstrated that changes in LVEF and WMSI are significantly associated with patient prognosis.

Echocardiography is commonly performed in AMI patients to assess structural and functional abnormalities following infarction. This examination provides numerous parameters, among which LVEF and WMSI are widely used by clinicians to evaluate patient prognosis due to their strong predictive value. LVEF and WMSI are influenced by various factors, including infarct size, symptom-to-balloon time, and clinical factors such as hypertension and diabetes. Nevertheless, they exhibit dynamic characteristics, as they can be modified through interventional and pharmacological treatments. Classifying patients based on changes in LVEF and WMSI revealed that, despite all AMI patients undergoing primary PCI, only 44.5% showed improved LV systolic function and alleviated WMAs one year after PCI compared to baseline. In contrast, 55.5% experienced worsening LV systolic function, WMAs, or both. These findings indicate that, despite primary reperfusion with PCI for AMI, changes in LVEF and WMSI are influenced by additional factors. Delaying revascularization and extension of myocardial infarct size may affect initial LV systolic function and WMAs in the acute phase of MI (22, 23). Additionally, primary revascularization of both infarct-related and non-infarct-related arteries, along with pharmacotherapy, influences LV systolic function and WMAs in the late phase of MI (24). This is consistent with our results. The random forest model identified symptom-to-balloon time, baseline LVEF and WMSI, coronary artery disease status, lesion characteristics in coronary vessels, imaging-guided PCI, and pharmacologic treatments such as beta-blockers and ACE inhibitors as factors associated with improvement in LV systolic function and WMAs (Supplementary Figure S6). In the baseline characteristics, patients who showed improvement in LVEF and WMSI had a significantly lower prevalence of hypertension, prior MI, prior revascularization, and NSTEMI. They also experienced a shorter symptom-to-balloon time and were more likely to receive beta-blockers and ACE inhibitors compared to other patients. Given the influence of these various factors, changes in LVEF and WMSI can differ among patients, even after undergoing PCI for AMI. Furthermore, these variations are highly likely to have impacted patient outcomes.

In multivariable logistic regression analysis, changes in LVEF and WMSI were identified as significant predictors of MACE, along with prior revascularization, history of MI, diabetes mellitus, hypertension, age, imaging-guided PCI, beta-blocker therapy, ACE inhibitor therapy. Additionally, we applied the random forest model to reduce overfitting, improve prediction accuracy, handle complex nonlinear relationships, and identify the most important predictive factors. Although feature importance may differ between the multivariable logistic regression model and the random forest model due to differences in how importance is measured, handling of collinearity, and ability to detect interactions, changes in LVEF and WMSI remained significant predictors of MACE in the random forest model as well.

The event rates of all-cause death, recurrent MI, rehospitalization for HF, repeat revascularization, and stent thrombosis during 3-year follow-up were significantly lower in patients with improvement in both LVEF and WMSI than others. In contrast, patients with worsened LVEF and WMSI had the highest event rates of all-cause death, recurrent MI, rehospitalization for HF, repeat revascularization and stent thrombosis. Furthermore, imaging-guided PCI and pharmacotherapy (beta-blockers and ACE inhibitors) are important predictive factors for MACE. These results suggest that a prerequisite for improving LV systolic function and WMAs in patients with AMI is to optimize stent implantation to ensure adequate blood flow to the ischemic myocardium and minimize stent-related complications. In contrast, suboptimal stent implantation causes inadequate blood flow to the ischemic myocardium and repetitive stent-related complications, resulting in progressive LV dysfunction and WMAs, adverse LV remodeling, and poor prognosis.

Furthermore, our study demonstrated that changes in LVEF and WMSI were predictive of LV remodeling. P. van der Bijl et al. categorized LV remodeling after AMI into early, mid-term, and late remodeling based on time points at 3, 6, and 12 months post-infarction (6). Additionally, several previous studies have reported the effects of pharmacological therapy (beta-blockers and ACE inhibitors) one year after myocardial infarction, observing changes in left ventricular end-systolic volume index, stroke volume index and ejection fraction, and the extent of LV dilation (24, 25). At one year, the remodeling process reaches a state of relative stability, making it the ideal time to evaluate the long-term effects of MI and therapeutic interventions on left ventricular function and structure. A decline in LVEF and/or WMSI was significantly associated with the development of adverse remodeling in this study. However, 12.2% of patients who improved LVEF and WMSI detected LV adverse remodeling. This is likely related to the definition of LV adverse remodeling. Several studies have defined post-infarct LV adverse remodeling as LV dilatation with a ≥20% increase in LV-end diastolic volume (LVEDV) compared to baseline value (6, 20). However, this definition has limitations in identifying all cases of LV adverse remodeling in AMI patients. Specifically, it may fail to detect patients who experience significant LV dilatation at baseline and throughout the 12-month follow-up period, even if they improve LVEF and WMSI. Therefore, further research is needed to refine the definition of LV adverse remodeling. The existing definitions of post-infarct LV adverse remodeling (LV dilatation, defined as a ≥20% increase in LVEDV from baseline) and LV reverse remodeling (a ≥15% reduction in LVESV and a ≥10% improvement in LVEF) are challenging for clinicians to calculate easily in real-world practice, limiting their practical application. This study defines the improvement in LV systolic function and WMAs as an increase in LVEF and a decrease in WMSI at 12 months compared to baseline, providing a simple and straightforward assessment of these changes. This method enables clinicians to quickly and easily calculate changes in LVEF and WMSI by simply subtracting the baseline values measured at admission from those recorded one year later.

Patients with improvement in both LVEF and WMSI had lower LV-end systolic volume (LVESV) and LV-end diastolic volume (LVEDV) at 12 months compared to others. This result supports the finding that patients with improvement in both LVEF and WMSI had a higher rate of reverse remodeling and a lower rate of adverse remodeling. Overall, our findings indicate that the best patient outcomes−characterized by the lowest rates of MACE and LV adverse remodeling−were observed in patients who showed improvement in both LVEF and RWMI. This suggests that LVEF and RWMI improvements might have a synergistic effect. Along with therapeutic strategies aimed at improving LVEF and WMSI, monitoring LVEF and WMSI over time is crucial for assessing ventricular remodeling after AMI. This approach may help prevent LV adverse remodeling and improve long-term prognosis.

Guideline-based optimal pharmacotherapy after PCI, including ACE inhibitors and beta-blockers, is essential to prevent adverse cardiovascular events or post-infarct LV adverse remodeling in patients with AMI. This is consistent with our findings. Beta-blockers reduce myocardial workload and oxygen demand by reducing the heart rate and blood pressure (26). They also help decrease catecholamine levels, myocardial ischemia, infarct size, incidence of fatal arrhythmia, sudden cardiac death, and early and late reinfarction (27, 28). However, the long-term benefits of beta-blocker therapy in patients with AMI and mildly reduced or preserved ejection fraction remain controversial (29). Our findings may help explain why beta-blockers failed to reduce primary outcomes (composite of death from any cause or new MI) in patients with an LVEF of at least 50% in the REDUCE-AMI trial (30). In our study, LVEF was negatively correlated with WMSI (WMSI = 2.78–0.03 × LVEF, Pearson's correlation coefficient = −0.73; *P* < 0.001) (Supplementary Figure S1). According to this linear regression equation, AMI patients with LVEF ≥50% had a WMSI of ≤1.28, suggesting smaller infarct sizes. In contrast, large infarcts (transmural MI) significantly impair LV function and worsen wall motion abnormalities (WMAs). However, our study demonstrated that the improvement in LV function and WMAs was most pronounced in patients with lower baseline LVEF and higher baseline WMSI. In AMI patients with small infarct size, the degree of change in LV function and WMAs may be minimal, and the likelihood of adverse LV remodeling is low if the stent implantation is optimized. Therefore, the effect of beta-blocker therapy may be minimal in these patients. However, as this estimation is derived from values calculated using a linear equation to represent the relationship between LVEF and WMSI, its interpretation should be approached with caution. Further research is needed to clarify the unresolved questions regarding beta-blocker therapy in AMI patients.

This study has several limitations that should be considered when interpreting its findings. First, as a retrospective study, the results are hypothesis-generating and should be generalized with caution. The observational nature of the study limits the ability to establish causal relationships, and potential confounding factors may influence the outcomes. Second, although intravascular imaging-guided PCI has been shown to be effective in patients with AMI (31, 32), it is often performed in hemodynamically stable patients, which may introduce selection bias. Third, this study used LVEF and WMSI as echocardiographic parameters to evaluate clinical outcomes after AMI. However, cardiac MR data, which is considered a key imaging modality for assessing LV remodeling, was not included. The absence of cardiac MR may limit the precision of the LV remodeling assessment. Fourth, In Korea, echocardiography is not routinely performed at 1 week, 1 month, or 6 months after myocardial infarction due to reimbursement policies. Therefore, as echocardiography data for these time points are unavailable, we could not assess LV remodeling during these periods. Fifth, patients who demonstrated improvement in both LVEF and WMSI had lower rates of hypertension, prior MI, and revascularization compared to other groups. Additionally, these patients had higher rates of beta-blocker and ACE inhibitor therapy, which may have contributed to the observed improvements in LV systolic function and WMAs after PCI. These differences in baseline characteristics could have influenced the study results. Finally, only patients who underwent echocardiography at both baseline and one year after AMI were included in this study. As a result, patients who did not complete both echocardiographic assessments or who experienced clinical events during this period were excluded. This exclusion criterion introduces survival bias, as patients with worse prognoses may have been underrepresented.

In conclusion, the degree of change in LVEF and WMSI differed among the patients with AMI who underwent PCI. This study also demonstrated that changes in LVEF and WMSI are significant predictors of clinical outcomes and LV remodeling in patients with AMI. Patients who exhibited improvement in both LVEF and WMSI had better long-term outcomes, including lower rates of MACE and LV adverse remodeling, compared to those with no improvement. These findings suggest that continuous monitoring of LVEF and WMSI post-PCI provide valuable prognostic insights. Optimizing stent implantation and pharmacological interventions, including beta-blockers and ACE inhibitors, may further enhance LV functional recovery. Implementing a tailored approach based on changes in LVEF and WMSI could improve patient management and long-term cardiovascular outcomes after AMI.

## Data Availability

The original contributions presented in the study are included in the article/Supplementary Material, further inquiries can be directed to the corresponding author.

## Supplementary material

Supplementary Figure S1 Correlation between LVEF and WMSI among patients with successful PCI due to AMI A scatter plot showed a negative correlation between baseline LVEF and WMSI (A). Changes in LVEF and WMSI were also negatively correlated between the baseline and 1-year follow-up after PCI (B). LVEF, left ventricular ejection fraction; WMSI, wall motion score index; PCI, percutaneous coronary intervention; AMI, acute myocardial infarction; Change in LVEF and WMSI was calculated by subtracting the values measured at initial hospitalization from those measured one year post-PCI.

Supplementary Figure S2 Inter-operator variability estimated using hospital-based differences The variance decomposition plot for LVEF and WMSI illustrates inter-operator variability. (A) represents the estimated variability at baseline, while (B) shows the estimation at the 12-month follow-up, both based on hospital-related differences. LVEF, left ventricular ejection fraction; WMSI, wall motion score index.

Supplementary Figure S3 Pairwise comparison of receiver operating characteristic (ROC) curves for LV reverse remodeling. The pairwise comparison of ROC curves for LV reverse remodeling is presented. LV, left ventricular; LVEF, left ventricular ejection fraction; WMSI, wall motion score index.

Supplementary Figure S4 Pairwise comparison of receiver operating characteristic (ROC) curves for LV adverse remodeling. Pairwise comparison of ROC curves for LV adverse remodeling in all patients (A), in patients with baseline LVEF >40% (B), and in patients with baseline LVEF ≤40% (C). LV, left ventricular; LVEF, left ventricular ejection fraction; WMSI, wall motion score index.

Supplementary Figure S5 Predicting factors for LV reverse remodeling. Random forest model (A) and logistic regression model (B) were utilized to identify predictors of LV reverse remodeling, and the results are presented. LVEF, left ventricular ejection fraction; WMSI, wall motion score index; BMI, body mass index; ACEi, Angiotensin-converting enzyme inhibitors; STEMI, ST-elevation myocardial infarction; NSTEMI, non-ST-elevation myocardial infarction; TIMI, thrombolysis in myocardial infarction; CAD, coronary artery disease; PCI, percutaneous coronary intervention; AHA/ACC, American Heart Association and American College of Cardiology.

Supplementary Figure S6 Predicting factors for improvement in LVEF and WMSI. The results of random forest model to identify predictors of LV adverse remodeling are presented. LVEF, left ventricular ejection fraction; WMSI, wall motion score index; BMI, body mass Supplementary Figure 6. Predicting factors for improvement in LVEF and WMSI The results of random forest model to identify predictors of LV adverse remodeling are presented. LVEF, left ventricular ejection fraction; WMSI, wall motion score index; BMI, body mass Supplementary Figure 6. Predicting factors for improvement in LVEF and WMSI The results of random forest model to identify predictors of LV adverse remodeling are presented. LVEF, left ventricular ejection fraction; WMSI, wall motion score index; BMI, body mass index; ACEi, Angiotensin-converting enzyme inhibitors; STEMI, ST-elevation myocardial infarction; NSTEMI, non-ST-elevation myocardial infarction; TIMI, thrombolysis in myocardial infarction; CAD, coronary artery disease; PCI, percutaneous coronary intervention; AHA/ACC, American Heart Association and American College of Cardiology.
