# Supplementary material for: Prognostic impact of changes in left ventricular ejection fraction and wall motion score index in patients with myocardial infarction
Source: Front Cardiovasc Med. 2025 May 14;12:1530006. doi: 10.3389/fcvm.2025.1530006 (PMC12116554; doi:10.3389/fcvm.2025.1530006)
Supplement: Supplementary Figure S6 — Predicting factors for improvement in LVEF and WMSI. The results of random forest model to identify predictors of LV adverse remodeling are presented. LVEF, left ventricular ejection fraction; WMSI, wall motion score index; BMI, body mass Supplementary Figure 6. Predicting factors for improvement in LVEF and WMSI The results of random forest model to identify predictors of LV adverse remodeling are presented. LVEF, left ventricular ejection fraction; WMSI, wall motion score index; BMI, body mass Supplementary Figure 6. Predicting factors for improvement in LVEF and WMSI The results of random forest model to identify predictors of LV adverse remodeling are presented. LVEF, left ventricular ejection fraction; WMSI, wall motion score index; BMI, body mass index; ACEi, Angiotensin-converting enzyme inhibitors; STEMI, ST-elevation myocardial infarction; NSTEMI, non-ST-elevation myocardial infarction; TIMI, thrombolysis in myocardial infarction; CAD, coronary artery disease; PCI, percutaneous coronary intervention; AHA/ACC, American Heart Association and American College of Cardiology. [file Datasheet1.pdf]

# Supplementary Materials

**Prognostic impact of changes in left ventricular systolic function and wall motion score index in patients with myocardial infarction**

**KS Lee et al.**

**Contents**

**#1. Supplementary Table 1.**

Univariate and Multivariate logistic regression analysis for improvement of LVEF and WMAs in patients with successful PCI due to AMI

**#2. Supplementary Table 2.**

Odd ratio (OR) for LV remodeling according to imaging-guided PCI and beta-blocker therapy

**#3. Supplementary Figure 1.**

Correlation between LVEF and WMSI among patients with successful PCI due to AMI

**#4. Supplementary Figure 2.**

Inter-operator variability estimated using hospital-based differences

**#5. Supplementary Figure 3.**

Pairwise comparison of ROC curves for predicting LV reverse remodeling

**#6. Supplementary Figure 4.**

Pairwise comparison of ROC curves for LV adverse remodeling

**#7. Supplementary Figure 5.**

Predicting factors for LV Reverse remodeling

**#8. Supplementary Figure 6.**

Predicting factors for improvement of LVEF and WMSI

**Supplementary Table 1. Univariate and Multivariate logistic regression analysis for improvement of LVEF and WMAs in patients with successful PCI due to AMI**

| Variables | Univariate analysis | | Multivariate analysis | |
|---|---|---|---|---|
| | HR (95% CI) | P-value | HR (95% CI) | P-value |
| **Patient factors** | | | | |
| Age (<65 years vs. ≥65) | 0.83 (0.71-0.98) | 0.026 | 0.91 (0.78-1.07) | 0.262 |
| Sex (female vs. male) | 1.05 (0.88-1.24) | 0.605 | | |
| Body mass index, kg/$m^2$ | 0.90 (0.76-1.06) | 0.220 | | |
| Hypertension | 0.87 (0.74-1.01) | 0.071 | | |
| Diabetes | 0.92 (0.77-1.11) | 0.387 | | |
| Dyslipidemia | 1.09 (0.85-1.40) | 0.517 | | |
| Prior myocardial infarction | 0.64 (0.46-0.90) | 0.010 | 0.65 (0.46-0.94) | 0.020 |
| Prior heart failure | 0.99 (0.42-2.35) | 0.980 | | |
| Smoking status | | | | |
| Never smoker | reference | | | |
| Ex-smoker | 1.11 (0.90-1.36) | 0.333 | | |
| Current smoker | 1.00 (0.85-1.17) | 0.951 | | |
| Killip classification | | | | |
| Class I | reference | | | |
| Class II | 1.08 (0.82-1.39) | 0.614 | | |
| Class III | 0.79 (0.59-1.08) | 0.142 | | |
| Class IV | 0.79 (0.50-1.24) | 0.307 | | |
| Family history of CAD | 0.95 (0.71-1.29) | 0.749 | | |
| Initial SBP (<130 vs≥130) | 1.31 (1.12-1.53) | 0.001 | 1.21 (1.04-1.42) | 0.017 |
| Initial HR (≤75 vs. >75) | 1.16 (0.99-1.35) | 0.072 | | |
| Anemia (<12mg/dL) | 0.84 (0.69-1.02) | 0.080 | | |
| LVEF, % (≤40% vs.> 40%) | 0.79 (0.65-0.97) | 0.025 | 0.93 (0.74-1.24) | 0.548 |
| LVESVI, mm2 (<23.8 vs. 23.8) | 1.05 (0.86-1.28) | 0.627 | | |
| Diagnosis | | | | |
| NSTEMI | Reference | | | |
| STEMI | 0.86 (0.73-1.00) | 0.057 | | |
| Initial LDL-C | | | | |
| Quartile 1 | Reference | | | |
| Quartile 2 | 0.96 (0.76-1.20) | 0.621 | | |
| Quartile 3 | 1.06 (0.85-1.33) | 0.730 | | |
| Quartile 4 | 1.24 (0.99-1.55) | 0.133 | | |
| Initial HDL-C | | | | |
| Quartile 1 | Reference | | | |
| Quartile 2 | 1.18 (0.94-1.49) | 0.154 | | |
| Quartile 3 | 1.07 (0.86-1.34) | 0.549 | | |
| Quartile 4 | 1.23 (0.98-1.55) | 0.072 | | |
| ACC/AHA lesion classification | | | | |
| Type A | Reference | | | |
| Type B1 | 2.03 (0.91-4.62) | 0.091 | | |
| Type B2 | 1.88 (0.84-4.19) | 0.123 | | |
| Type C | 1.73 (0.78-3.85) | 0.176 | | |
| Initial TIMI flow | | | | |
| Grade 3 | Reference | | Reference | |
| Grade 2 | 0.92 (0.68-1.14) | 0.325 | 0.95 (0.72-1.24) | 0.700 |
| Grade 1 | 1.03 (0.78-1.35) | 0.862 | 1.01 (0.76-1.36) | 0.937 |
| Grade 0 | 0.73 (0.61-0.89) | 0.002 | 0.69 (0.57-0.85) | <0.001 |
| **Procedure or Post PCI factors** | | | | |

| | | | | |
|---|---|---|---|---|
| Prasugrel or ticagrelor during 1Y | 0.80 (0.63-1.03) | 0.085 | | |
| ACE inhibitor during 1Y | 1.33 (1.13-1.57) | 0.001 | 1.32 (1.07-1.62) | 0.008 |
| Basal statin | 1.30 (0.96-1.78) | 0.955 | | |
| Imaging-guided PCI | 1.17 (0.99-1.40) | 0.070 | | |
| Imaging-guided PCI and BB therapy for 1Y | | | | |
| Angiography-guided PCI and non-BB treatment | Reference | | Reference | |
| Imaging-guided PCI without BB therapy or Angiography-guided PCI and BB therapy | 1.24 (1.03-1.49) | 0.026 | 1.27 (1.02-1.57) | 0.031 |
| Imaging-guided PCI and BB therapy | 1.53 (1.20-1.93) | <0.001 | 1.46 (1.12-1.90) | 0.005 |
| Stent diameter | | | | |
| Quartile 1 | Reference | | | |
| Quartile 2 | 0.93 (0.77-1.14) | 0.586 | | |
| Quartile 3 | 1.12 (0.91-1.37) | 0.511 | | |
| Quartile 4 | 0.97 (0.73-1.29) | 0.828 | | |
| Stent length | | | | |
| Quartile 1 | Reference | | | |
| Quartile 2 | 1.27 (1.02-1.58) | 0.035 | 1.16 (0.93-1.43) | 0.190 |
| Quartile 3 | 1.08 (0.86-1.35) | 0.501 | 1.01 (0.81-1.26) | 0.953 |
| Quartile 4 | 1.14 (0.91-1.43) | 0.270 | 1.02 (0.81-1.27) | 0.892 |
| Post PCI TIMI flow | | | | |
| Grade 3 | Reference | | | |
| Grade 2 | 0.68 (0.44-1.06) | 0.097 | | |
| Grade 1 | 0.83 (0.22-3.23) | 0.792 | | |
| PCI to LM | 1.08 (0.69-1.69) | 0.738 | | |
| PCI to LAD | 1.31 (1.02-1.68) | 0.032 | 0.87 (0.73-1.05) | 0.147 |
| PCI to LCX | 0.96 (0.82-1.12) | 0.576 | | |
| PCI to RCA | 0.90 (0.77-1.04) | 0.213 | | |
| Revascularization strategy | | | | |
| Culprit-lesion-only PCI | Reference | | | |
| Multivessel PCI | 0.99 (0.82-1.20) | 0.966 | | |
| PCI procedures | | | | |
| Index procedure PCI | Reference | | | |
| Staged PCI | 0.82 (0.64-1.05) | 0.109 | | |

Abbreviations: HR, hazard ratio; CI, confidence interval; CAD, coronary artery disease; LVEF, left ventricular ejection fraction; NSTEMI, non-ST elevation myocardial infarction; STEMI, ST elevation myocardial infarction; LDL-C, low-density lipoprotein cholesterol; HDL-C, high-density lipoprotein cholesterol; ACC/AHA, American college of cardiology/American heart association; TIMI, thrombolysis in myocardial infarction; PCI, percutaneous coronary intervention; RAAS, renin-angiotensin-aldosterone system; FFR, fraction flow reserve; BB, beta-blocker; LM, left main; LAD, left anterior descending; LCX, left circumflex; RCA, right coronary artery

**Supplementary Table 2. Odd ratio (OR) for LV remodeling according to imaging-guided PCI and beta-blocker therapy**

| LV remodeling | ¶Adjusted OR (95% CI) | *P*-value |
|---|---|---|
| **[a]LV reverse remodeling in patients with baseline LVEF> 40%** | | |
| Angiography-guided PCI without BB therapy | 1 | |
| Imaging-guided PCI without BB therapy | 1.05 (0.78-1.41) | 0.765 |
| Angiography-guided PCI with BB therapy | 0.88 (0.59-1.30) | 0.522 |
| Imaging-guided PCI with BB therapy | 1.10 (0.79-1.54) | 0.573 |
| **[b]LV adverse remodeling in patients with baseline LVEF> 40%** | | |
| Angiography-guided PCI without BB therapy | 1 | |
| Imaging-guided PCI without BB therapy | 1.05 (0.75-1.46) | 0.781 |
| Angiography-guided PCI with BB therapy | 1.02 (0.67-1.57) | 0.917 |
| Imaging-guided PCI with BB therapy | 1.10 (0.76-1.60) | 0.608 |
| **LV reverse remodeling in patients with baseline LVEF≤ 40%** | | |
| Angiography-guided PCI without BB therapy | 1 | |
| Imaging-guided PCI without BB therapy | 1.57 (0.77-3.17) | 0.213 |
| Angiography-guided PCI with BB therapy | 1.85 (0.48-7.19) | 0.375 |
| Imaging-guided PCI with BB therapy | 4.29 (1.67-10.97) | 0.002 |
| **LV adverse remodeling in patients with baseline LVEF≤ 40%** | | |
| Angiography-guided PCI without BB therapy | 1 | |
| Imaging-guided PCI without BB therapy | 0.61 (0.29-1.27) | 0.187 |
| Angiography-guided PCI with BB therapy | 0.72 (0.21-2.44) | 0.603 |
| Imaging-guided PCI with BB therapy | 0.64 (0.36-1.14) | 0.066 |

Abbreviations: LV, left ventricular; PCI, percutaneous coronary intervention; BB, beta-blocker; LVEF, left ventricular ejection fraction

¶Adjusted variables: age, sex, BMI, hypertension, diabetes mellitus, previous MI, baseline blood pressure, angiotensin-converting enzyme (ACE) inhibitor treatment.

a: LV post-infarct adverse remodeling was defined as LV dilatation with LVEDV increase of ≥20% from baseline to 1-year follow-up after PCI

b: LV reverse remodeling was defined as ≥15% reduction in LVESV from baseline to 1-year follow-up after PCI

**Supplementary Figure 1.Correlation between LVEF and WMSI among patients with successful PCI due to AMI**

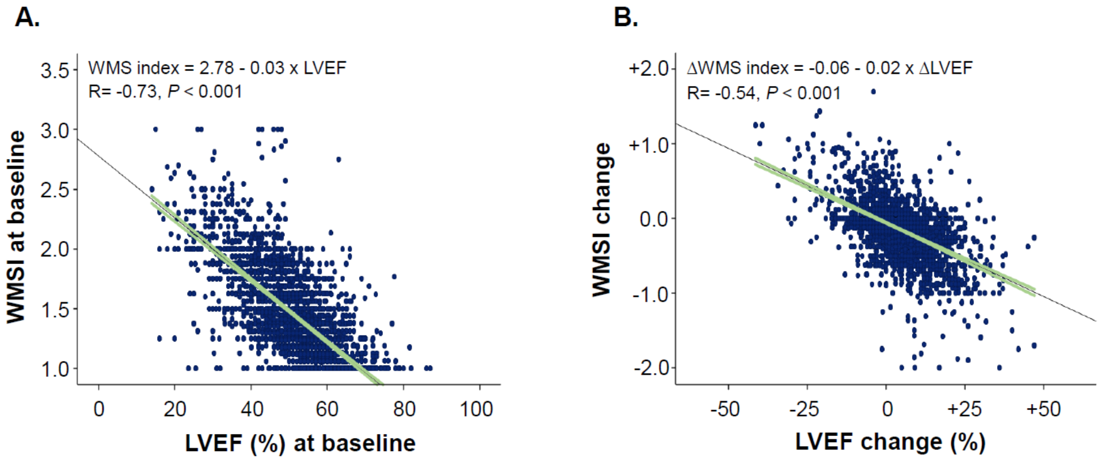


A scatter plot showed a negative correlation between baseline LVEF and WMSI (A). Changes in LVEF and WMSI were also negatively correlated between the baseline and 1-year follow-up after PCI (B).

LVEF, left ventricular ejection fraction; WMSI, wall motion score index; PCI, percutaneous coronary intervention; AMI, acute myocardial infarction; Change in LVEF and WMSI was calculated by subtracting the values measured at initial hospitalization from those measured one year post-PCI.

**Supplementary Figure 2. Inter-operator variability estimated using hospital-based differences**

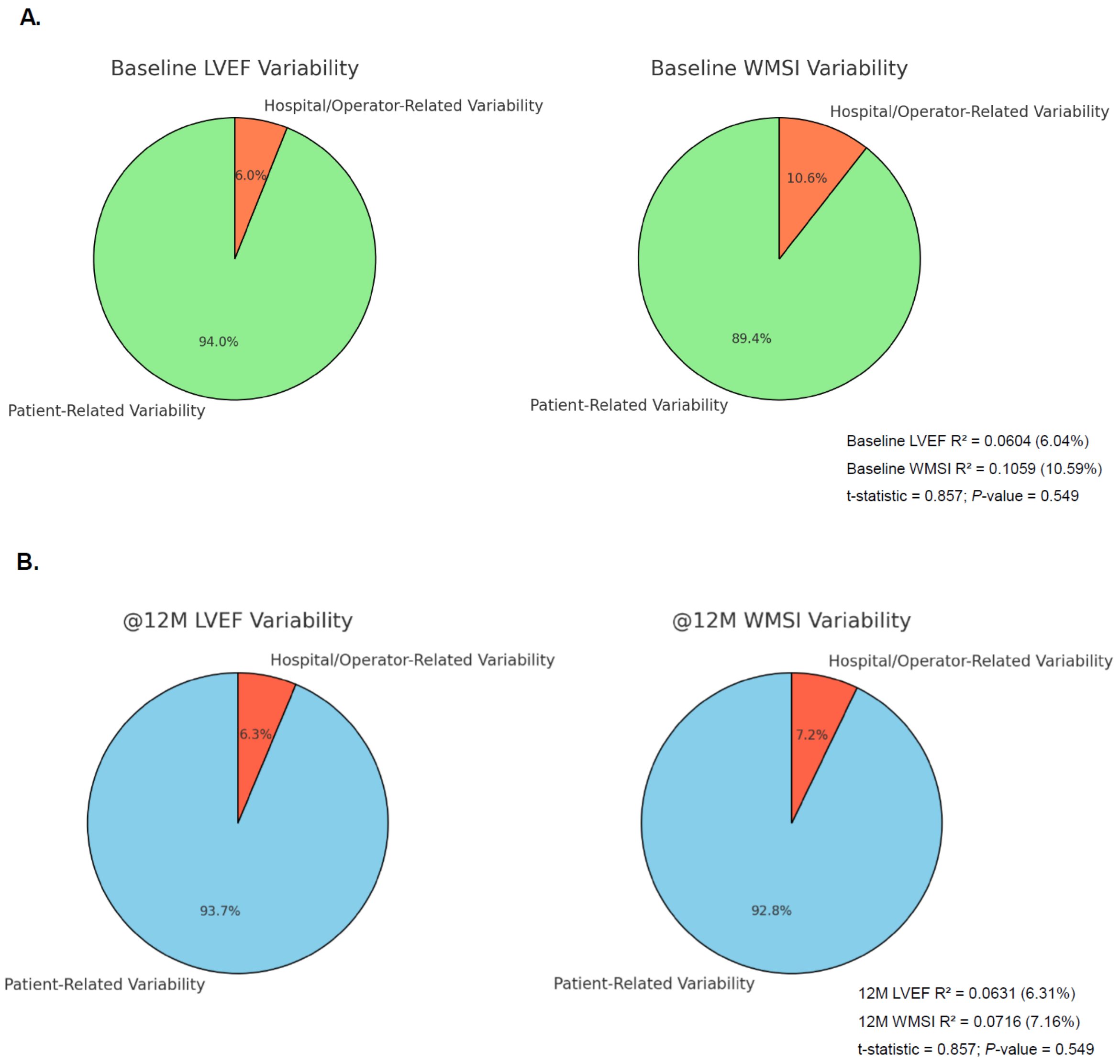


The variance decomposition plot for LVEF and WMSI illustrates inter-operator variability. (A) represents the estimated variability at baseline, while (B) shows the estimation at the 12-month follow-up, both based on hospital-related differences.

LVEF, left ventricular ejection fraction; WMSI, wall motion score index

**Supplementary Figure 3. Pairwise comparison of ROC curves for predicting LV reverse remodeling**

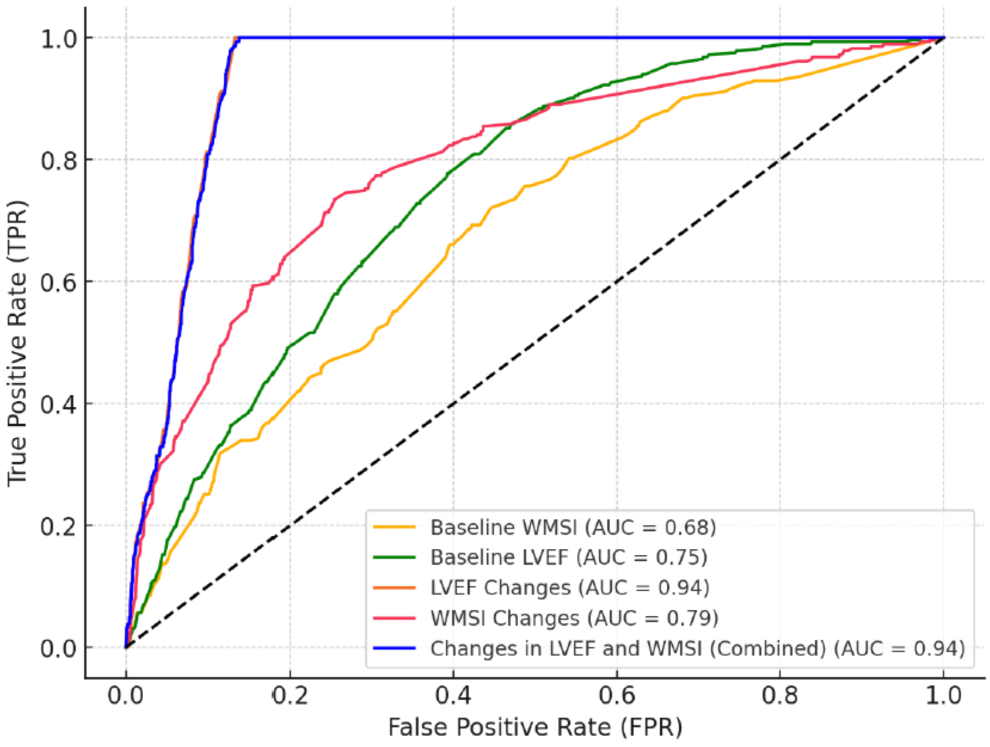


The pairwise comparison of ROC curves for LV reverse remodeling is presented.

LV, left ventricular; LVEF, left ventricular ejection fraction; WMSI, wall motion score index

**Supplementary Figure 4. Pairwise comparison of ROC curves for LV adverse remodeling**

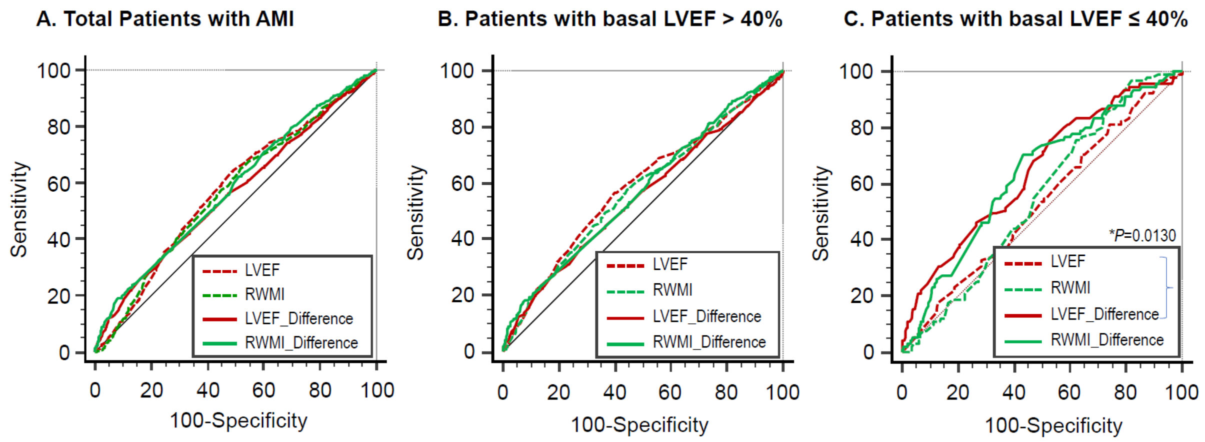


| Variable | AUC | SE | 95% CI |
|---|---|---|---|
| Baseline LVEF | 0.58 | 0.0141 | 0.55-0.60 |
| Baseline WMSI | 0.57 | 0.0141 | 0.55-0.59 |
| △LVEF | 0.56 | 0.0146 | 0.54-0.58 |
| △WMSI | 0.58 | 0.0141 | 0.56-0.60 |

| Variable | AUC | SE | 95% CI |
|---|---|---|---|
| Baseline LVEF | 0.59 | 0.0159 | 0.56-0.61 |
| Baseline WMSI | 0.57 | 0.0159 | 0.55-0.60 |
| △LVEF | 0.55 | 0.0162 | 0.53-0.57 |
| △WMSI | 0.57 | 0.0156 | 0.55-0.60 |

| Variable | AUC | SE | 95% CI |
|---|---|---|---|
| Baseline LVEF | 0.53 | 0.0354 | 0.47-0.59 |
| Baseline WMSI | 0.55 | 0.0334 | 0.50-0.61 |
| △LVEF | 0.65 | 0.0341 | 0.59-0.70 |
| △WMSI | 0.63 | 0.0340 | 0.58-0.69 |

Pairwise comparison of ROC curves for LV adverse remodeling in all patients (A), in patients with baseline LVEF >40% (B), and in patients with baseline LVEF ≤40% (C).

LV, left ventricular; LVEF, left ventricular ejection fraction; WMSI, wall motion score index

**Supplementary Figure 5. Predicting factors for LV Reverse remodeling**

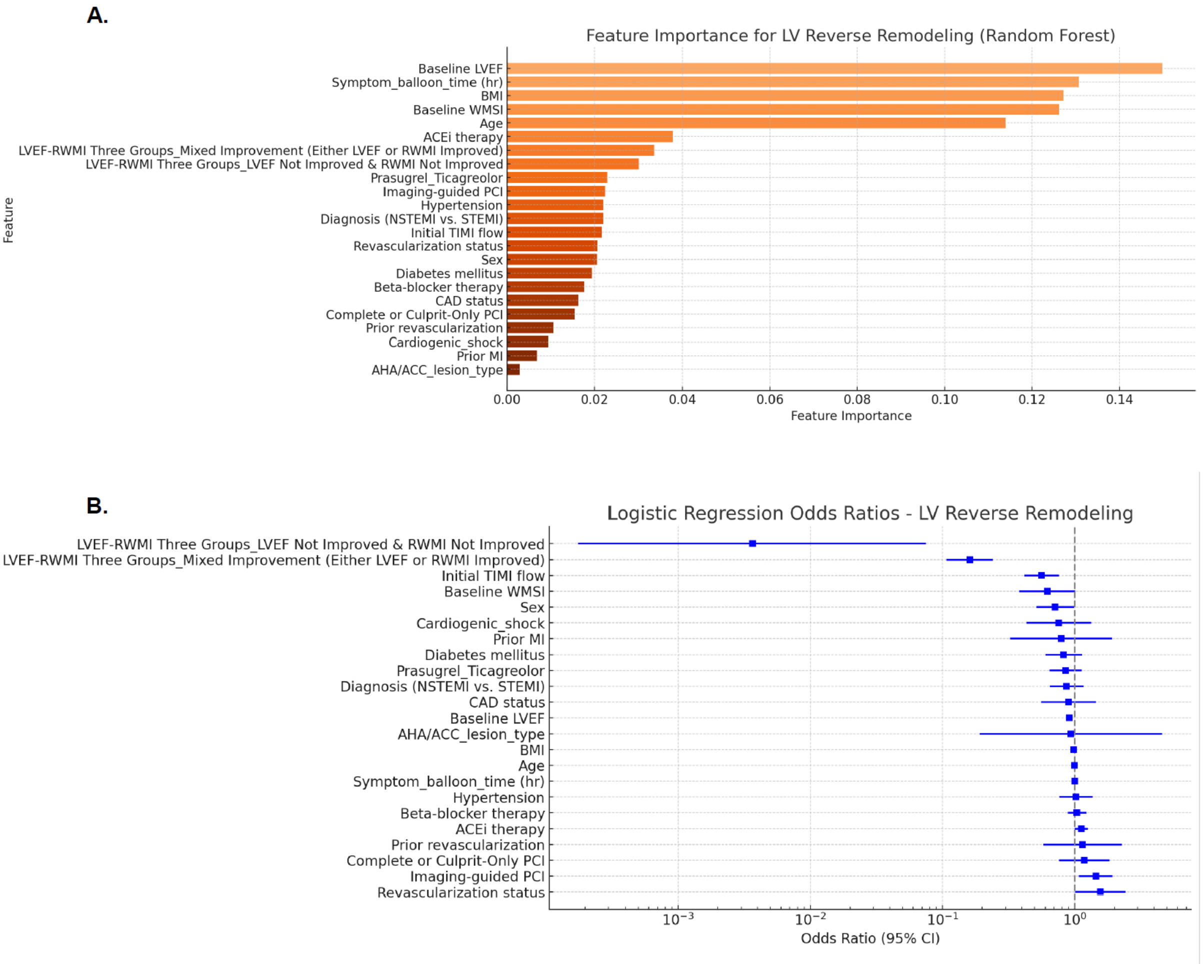


Random forest model (A) and logistic regression model (B) were utilized to identify predictors of LV reverse remodeling, and the results are presented.

LVEF, left ventricular ejection fraction; WMSI, wall motion score index; BMI, body mass index; ACEi, Angiotensin-converting enzyme inhibitors; STEMI, ST-elevation myocardial infarction; NSTEMI, non-ST-elevation myocardial infarction; TIMI, thrombolysis in myocardial infarction; CAD, coronary artery disease; PCI, percutaneous coronary intervention; AHA/ACC, American Heart Association and American College of Cardiology.

**Supplementary Figure 6. Predicting factors for improvement of LVEF and WMSI**

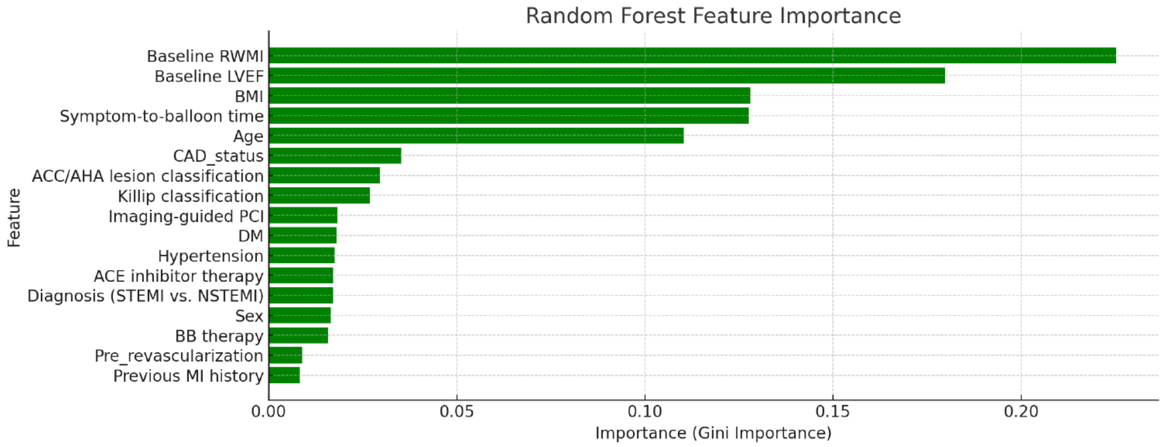


The results of random forest model to identify predictors of LV adverse remodeling are presented.

LVEF, left ventricular ejection fraction; WMSI, wall motion score index; BMI, body mass index; ACEi, Angiotensin-converting enzyme inhibitors; STEMI, ST-elevation myocardial infarction; NSTEMI, non-ST-elevation myocardial infarction; TIMI, thrombolysis in myocardial infarction; CAD, coronary artery disease; PCI, percutaneous coronary intervention; AHA/ACC, American Heart Association and American College of Cardiology
